# Complete Unique Genome Sequence, Expression Profile, and Salivary Gland Tissue Tropism of the Herpesvirus 7 Homolog in Pigtailed Macaques

**DOI:** 10.1128/JVI.00651-16

**Published:** 2016-07-11

**Authors:** Jeannette P. Staheli, Michael R. Dyen, Gail H. Deutsch, Ryan S. Basom, Matthew P. Fitzgibbon, Patrick Lewis, Serge Barcy

**Affiliations:** a Center for Global Infectious Disease Research, Seattle Children's Research Institute, Seattle, Washington, USA; b Pathology, Seattle Children's Hospital, Seattle, Washington, USA; c Genomics, Fred Hutchinson Cancer Research Center, Seattle, Washington, USA; d Computational Biology, Fred Hutchinson Cancer Research Center, Seattle, Washington, USA; e Department of Pediatrics, University of Washington, Seattle, Washington, USA; Northwestern University

## Abstract

Human herpesvirus 6A (HHV-6A), HHV-6B, and HHV-7 are classified as roseoloviruses and are highly prevalent in the human population. Roseolovirus reactivation in an immunocompromised host can cause severe pathologies. While the pathogenic potential of HHV-7 is unclear, it can reactivate HHV-6 from latency and thus contributes to severe pathological conditions associated with HHV-6. Because of the ubiquitous nature of roseoloviruses, their roles in such interactions and the resulting pathological consequences have been difficult to study. Furthermore, the lack of a relevant animal model for HHV-7 infection has hindered a better understanding of its contribution to roseolovirus-associated diseases. Using next-generation sequencing analysis, we characterized the unique genome of an uncultured novel pigtailed macaque roseolovirus. Detailed genomic analysis revealed the presence of gene homologs to all 84 known HHV-7 open reading frames. Phylogenetic analysis confirmed that the virus is a macaque homolog of HHV-7, which we have provisionally named Macaca nemestrina herpesvirus 7 (MneHV7). Using high-throughput RNA sequencing, we observed that the salivary gland tissue samples from nine different macaques had distinct MneHV7 gene expression patterns and that the overall number of viral transcripts correlated with viral loads in parotid gland tissue and saliva. Immunohistochemistry staining confirmed that, like HHV-7, MneHV7 exhibits a natural tropism for salivary gland ductal cells. We also observed staining for MneHV7 in peripheral nerve ganglia present in salivary gland tissues, suggesting that HHV-7 may also have a tropism for the peripheral nervous system. Our data demonstrate that MneHV7-infected macaques represent a relevant animal model that may help clarify the causality between roseolovirus reactivation and diseases.

**IMPORTANCE** Human herpesvirus 6A (HHV-6A), HHV-6B, and HHV-7 are classified as roseoloviruses. We have recently discovered that pigtailed macaques are naturally infected with viral homologs of HHV-6 and HHV-7, which we provisionally named MneHV6 and MneHV7, respectively. In this study, we confirm that MneHV7 is genetically and biologically similar to its human counterpart, HHV-7. We determined the complete unique MneHV7 genome sequence and provide a comprehensive annotation of all genes. We also characterized viral transcription profiles in salivary glands from naturally infected macaques. We show that broad transcriptional activity across most of the viral genome is associated with high viral loads in infected parotid glands and that late viral protein expression is detected in salivary duct cells and peripheral nerve ganglia. Our study provides new insights into the natural behavior of an extremely prevalent virus and establishes a basis for subsequent investigations of the mechanisms that cause HHV-7 reactivation and associated disease.

## INTRODUCTION

Human herpesvirus 7 (HHV-7) was first identified in 1990 as a lymphotropic virus belonging to the Roseolovirus genus within the Betaherpesvirinae subfamily (1). HHV-7 is one of the most prevalent viruses in the human population (2). Serological data suggest that primary infection occurs early in childhood and results in a lifelong infection. While primary HHV-7 infection can result in benign illness, like exanthem subitum (3), viral reactivation in immunosuppressed patients has been associated with severe pathological conditions (4, 5). Complications associated with virus reactivation include a wide range of diseases, including neurological pathologies (6).

When viral persistence following primary infection is established, the presence of detectable infectious virus particles in saliva is a common occurrence (7, 8). Both labial and submandibular salivary glands have been shown to be the anatomical sites for HHV-7 persistence, replication, and late viral protein expression (9). Within the gland tissue, virus seems to be predominantly localized in the ductal cuboidal and columnar cells. Thus, salivary glands are a likely source for infectious virus shed into saliva.

Complete genome sequences are available for all three human roseoloviruses, HHV-6A, HHV-6B, and HHV-7. For HHV-7, the genomes of three different strains, JI, RK, and UCL-1, have been sequenced using viral DNA isolated from peripheral blood mononuclear cells (PBMCs) (JI and RK) (1, 10) or saliva (UCL-1) (11, 12). Similar to other roseolovirus genomes, the genome structure of HHV-7 is composed of a central unique (U) 133-kb-long segment flanked by 10-kb-long end-terminal direct repeat (DR) regions on each side. Viral genes in the unique segment are arranged in blocks of genes conserved among herpesviruses and betaherpesviruses or are unique to roseoloviruses.

The HHV-7 origin of lytic replication (oriLyt) is located upstream of the major DNA-binding protein gene U41, similar to its location in other Betaherpesvirinae subfamily genomes. The oriLyt sequence contains two binding sites, origin-binding protein 1 (OBP-1) and OBP-2 (13), both of which are recognized by an origin-binding protein (OBP) encoded by the viral open reading frame (ORF) U73 (14). Interaction between OBP and the binding sites is required to initiate viral DNA replication. The HHV-7 unique region also contains two repetitive sequence motifs, R1 and R2 (15). The R1 segment is located between ORFs U86 and U89 and, in the RK strain, is composed of two 84-bp repeats plus two partial 67-bp repeats, with the nucleotide sequences being conserved among HHV-7 strains. The second segment, R2, is located downstream of R1 between ORFs U91 and U95 and is composed of 105-bp motifs repeated 16 times. The coding potential of the R1 and R2 repeat regions for either short proteins or noncoding RNAs is uncertain, and both regions have been shown to vary in size and sequence among different strains of HHV-7. The HHV-7 genome is predicted to encode 82 different proteins in the unique region, with their ORFs being annotated U2 to U100. In addition, two protein-coding ORFs (DR1 and DR6) have been identified in each of the terminal repeat regions. All but one of the ORFs in the HHV-7 genome have counterparts among the other roseolovirus genomes. The exception is U55B, which is found only in different HHV-7 strains and not in either HHV-6A or HHV-6B. Conversely, the HHV-7 genome lacks ORFs U22 and U94, which are present in both HHV-6 species. Some additional small expressed ORFs are present in the HHV-6B DR region but not in HHV-7.

Little is known about HHV-7 transcriptional activity in infected cells. One study reported that HHV-7 temporal gene expression is regulated by a cascade mechanism similar to that used for HHV-6 transcription. Interestingly, transcripts from four viral genes that had been determined to be immediate early genes in that study could not be detected by reverse transcription (RT)-quantitative real-time PCR (qPCR) assays in PBMCs from healthy patients with detectable levels of HHV-7 DNA (16). This observation suggests that PBMC infection with HHV-7 may result in latency with very limited transcriptional activity.

Recently, we and others have reported the existence of HHV-7 homologs naturally infecting different nonhuman primate species, including macaques (17) and great apes (18). All simian homologs of HHV-7 were discovered using consensus-degenerate hybrid oligonucleotide primers (CODEHOP) (19). This PCR-based approach led to the discovery of genomic segments comprised of the glycoprotein B (gB) and the DNA polymerase genes. Since our study used DNA from saliva samples collected from pigtailed macaques (Macaca nemestrina), we proposed to provisionally name the macaque HHV-7 homolog Macaca nemestrina herpesvirus 7 (MneHV7). Phylogenetic analysis based on the available sequence segments confirmed the close evolutionary relationship between the newly identified simian roseoloviruses and HHV-7. We further characterized the prevalence and tissue tropism of MneHV7 natural infection in pigtailed macaques by using a newly developed qPCR assay specific for MneHV7. We detected MneHV7 at high levels in all saliva samples screened and observed the highest viral loads in salivary gland tissues. Finally, we showed a strong correlation between viral loads in saliva and salivary glands. Altogether, these results are in agreement with previous observations made for HHV-7 in humans that salivary glands are likely a major site of MneHV7 replication and persistence.

Here we report the complete sequence of the unique segment of the MneHV7 genome as well as a portion of the 10-kb DR region at the genomic termini. A comparison of the MneHV7 genome with the HHV-7 genome revealed a colinearity of gene structure and a strong gene sequence similarity, confirming that MneHV7 is an HHV-7 homolog naturally infecting pigtailed macaques. We present a first annotation of the MneHV7 genome, based on similarity with the HHV-7 RK strain sequence. Genomic analysis of sequence elements crucial for roseolovirus biology suggests that MneHV7 relies on mechanisms similar to those relied on by HHV-7 to control its life cycle. Using high-throughput RNA sequencing (RNA-seq), we detected high-level expression of a broad range of MneHV7 genes in salivary gland tissue from naturally infected macaques. In addition, using immunohistochemistry (IHC), we observed late viral protein expression in salivary gland tissues, which correlated with high levels of MneHV7 gene expression and viral loads in saliva. Our study revealed that MneHV7 exhibits numerous molecular and biological similarities with HHV-7, demonstrating that natural infection with MneHV7 is a useful animal model to study HHV-7 infection and associated pathologies.

## MATERIALS AND METHODS

### Pigtailed macaque specimens.

Saliva and salivary gland tissue samples from 12 pigtailed macaques were obtained from stored aliquots collected in previous studies (20). All 12 animals had been part of a prior simian immunodeficiency virus (SIV) vaccine study. During saliva collection, the animals were kept under deep sedation with ketamine HCl at a dose of 10 to 15 mg/kg of body weight intramuscularly to alleviate any pain and discomfort. The animals were monitored by an animal technician or veterinary technologist while they were under sedation. Tissues collected at necropsy were either snap-frozen or fixed in 10% neutral buffered formalin and embedded in paraffin. All animals in this study were housed and cared for according to the *Guide for the Care and Use of Laboratory Animals* (21) at the Washington National Primate Research Center (WaNPRC), an Association for Assessment and Accreditation of Laboratory Animal Care International-accredited institution. The experimental procedures were approved by the Institutional Animal Care and Use Committee (2370-20) at the University of Washington and conducted in compliance with the guidelines in *Public Health Service Policy on Humane Care and Use of Laboratory Animals* (http://grants.nih.gov/grants/olaw/references/PHSPolicyLabAnimals.pdf).

### DNA isolation from saliva and tissues and library preparation.

DNA isolation from macaque saliva and tissue samples was performed as previously described (16) using proteinase K treatment at 56°C and a QIAamp DNA minikit according to the manufacturer's protocol (Qiagen). The DNA template used for next-generation sequencing was isolated from cell-free filtered saliva following a procedure similar to that described above but with an additional purification and concentration step using a DNA clean and concentrator spin column (Zymo Research). The sequencing library was prepared from sheared DNA using a TruSeq DNA sample preparation kit (ver2; Illumina, Inc.). The library size distribution was validated using an Agilent 2200 TapeStation instrument (Agilent Technologies).

### MneHV7-specific TaqMan qPCR assay.

The PCR primers and TaqMan probe used for quantitative real-time PCR (qPCR) assays specifically targeting the MneHV7 DNA *pol* gene were described before (16). Primer and probe sequences were as follows: MneHV7-pol probe, 5′-6FAM-ACTGGTGCAACACATAGCTTATTACCGT-BHQ1-3′ (where 6FAM represents 6-carboxyfluorescein and BHQ1 represents black hole quencher 1 dye); MneHV7-pol-F, 5′-GTGCAAAGACCCTACGTTAATTATG-3′; and MneHV7-pol-R, 5′-CTTGTTACCGAAGCAGCAATAG-3′. The thermocycling conditions were as follows: incubation at 94°C for 2.5 min, followed by 45 cycles of 94°C for 30 s, 60°C for 30 s, and 72°C for 30 s. The reaction mixture was then cooled for 1 min at 37°C. All steps were performed at a maximum ramp speed of 4.4°C/s. Standardized MneHV7 templates consisted of the purified plasmid pJET vector containing the cloned MneHV7 PCR amplicon. The viral load was determined by comparing the cycle threshold (*C_T_*) values obtained from the MneHV7 qPCR assay with the *C_T_* values obtained from a single-copy cellular gene, oncostatin M (OSM), as previously described (16).

### Next-generation sequencing and *de novo* assembly.

Library clustering was performed on a single flow cell lane using an Illumina cBot automated clonal amplification system, and sequencing subsequently occurred on an Illumina HiSeq 2500 sequencer using a paired-end, 50-nucleotide-read-length (PE50) strategy. Image analysis and base calling were performed using Illumina's Real Time Analysis (RTA; ver1.17) software, followed by the generation of FASTQ files, using Illumina's CASAVA (ver1.8.2) software.

Because the sample was from saliva, we expected a predominance of short reads from host DNA and sought to suppress these before attempting *de novo* assembly. Since a Macaca nemestrina reference genome was not available, preliminary alignment to the rhesus macaque genome (Ensembl MMUL 1.0) was used to filter out reads derived from the host. Approximately 215 million raw read pairs, all passing default quality filtering in the Illumina CASAVA (ver1.8.2) pipeline, were aligned to the rhesus macaque reference genome using the BWA (ver0.6.1) software package (19). This process classified nearly 83% of the input reads as aligning to and therefore originating from the host. Over 36 million read pairs that did not align to the rhesus macaque genome were assembled with the Velvet (ver1.2.08) algorithm (22). The resulting contigs were searched for loose matches to our previously determined MneHV7 DNA polymerase sequence (16). A match was found, and the properties of the matching contig were used to further optimize *de novo* assembly parameters and extend coverage. A single large contig of ∼122 kb, a significant portion of the expected ∼150-kb genome, was identified. Alignment of all raw Illumina reads against this candidate contig was then performed with BWA software, resulting in a collection of 34,571 read pairs which were used to further refine the assembly. Two additional consecutive short contigs of 6.8 kb and 2.4 kb were found, resulting in complete coverage of the expected length of the unique (U) genome region of MneHV7 and about one-quarter (2.4 kb of an estimated 10 kb) of the end-terminal direct repeat regions.

### Long-range sequencing.

The R2 repeat region was resolved by long-range PCR and Sanger sequencing using a TaKaRa LA PCR kit (ver2.1) following the manufacturer's protocol (Clontech). PCRs were performed using the following primer combinations: R2_1F_IDT (5′-CTCTCTTCAGTTGATGCCATAGT-3′) and R2_1R_IDT (5′-TTGCGAATCCCTGTCATAGTT-3′), R2_3F_IDT (5′-CAGTTCCATAGACAGAGCTAACA-3′) and R2_3R_IDT (5′-GGTGTGGGTCGAATAACATTTG-3′), and R2_4F_IDT (5′-CTGGATGGTTGTCCAACTACA-3′) and R2_4R_IDT (5′-AGGCTGATGAATACTGCTGAC-3′). The PCR buffer consisted of the LA PCR buffer II (10×, Mg; TaKaRa) supplemented with 5% dimethyl sulfoxide. Amplification conditions were as follows: one cycle of 94°C for 1 min, followed by 35 cycles of 98°C for 10 s, 58°C for 1 min, and 68°C for 5 min and one final cycle at 72°C for 10 min.

### Gene annotations.

The annotation of the MneHV7 genome was performed using the Genome Annotation Transfer Utility (GATU) provided at the Viral Bioinformatics Resource Center (23) and was fine-tuned using the Geneious software package (ver6.1.6; Biomatters). Open reading frames were verified by observing the presence and locations of start and stop codons as well as polyadenylation signal sequences in comparative alignments of the candidate MneHV7 sequence with the HHV-7 RK strain sequence (GenBank accession number NC_001716) using the various aligners (Geneious aligner, ClustalW) that are part of the Geneious software package. Predicted intron-exon boundaries of spliced genes in the HHV-7 RK sequence were detected in the MneHV7 genome sequence by locating conserved splice acceptor and donor sites in the sequence alignment.

### Comparative sequence and phylogenetic analysis.

Sequence analysis of specific regions of the MneHV7 genome was performed by alignment with known betaherpesvirus sequences using the Muscle (for amino acid sequences) and the Geneious (for nucleotide sequences) alignment algorithms, which are part of the Geneious software package (Biomatters). Phylogenetic analysis was performed using the maximum likelihood method (PhyML) with the JC69 substitution model (24) from the Muscle or Geneious alignments of herpesvirus amino acid sequences. The number of substitution rate categories was set at 4, and the best of both nearest neighbor interchange (NNI) and subtree pruning and regrafting (SPR) topology searches was combined into the final tree calculation. The percent likelihood of branching within the phylogenetic tree was calculated by using the approximate likelihood ratio test, which returned chi-square-based parametric branch supports (68).

The following viral DNA polymerase sequences were used to construct the phylogenetic tree and are deposited in GenBank under the indicated accession numbers: HHV-5/human cytomegalovirus (hCMV), YP_081513; Mandrillus herpesvirus 1β (MndHVbeta), AAG39066, HHV-6A strain U1102, CAA58372; HHV-6B strain Z29, AAD49652; Pan troglodytes roseolovirus 1 (Ptro roseolovirus 1), AAW55996; HHV-7 strain RK, AAC40752; HHV-7 strain JI, AAC54700; P. troglodytes herpesvirus 7 (PtroHV7), AIN81106; Pan paniscus herpesvirus 7 (PpanHV7), AIN81107, Gorilla gorilla herpesvirus 7 (GgorHV7) AIN81109; and MneHV7, KU351741.

### Immunohistochemistry.

Immunohistochemical staining was performed on paraffin-embedded sections after an endogenous peroxidase block with 3% H_2_O_2_ in methanol, antigen retrieval with citrate buffer, pH 6.0, a 5% normal serum block, and an avidin-biotin block (Vector Laboratories). The sections were incubated with KR4 primary antibody (1:1,000; catalog number 12179; NIH AIDS Reagent Program, Division of AIDS, NIAID, NIH) for 2 h at room temperature, followed by incubation with biotinylated anti-mouse immunoglobulin antibody (1:500) and a streptavidin-biotin-peroxidase complex (both from Vector Laboratories). Peroxidase activity was visualized using diaminobenzidine (Vector Laboratories). All histological stainings were repeated on 3 separate sections. Negative controls consisted of sections incubated without primary antibody and a matched isotype control. VirArray HHV-7 control cell slides (VAH7-05; Bioworld Consulting Laboratories) were used as a positive control.

### RNA isolation from salivary gland tissues.

Frozen salivary gland tissue specimens were minced with a sterile razor blade before addition of the TRIzol reagent. Following a 30-min incubation on ice, total RNA was isolated using a Direct-zol RNA miniprep column (Zymo Research) following the manufacturer's protocol. RNA samples were further purified on Clean and Concentrate columns (Zymo Research) according to the manufacturer's instructions. Total RNA integrity was checked using the Agilent 2200 TapeStation instrument (Agilent Technologies) and quantified using a Trinean DropSense96 spectrophotometer (Caliper Life Sciences).

### Viral transcriptome.

RNA-seq libraries were prepared from total RNA using a TruSeq RNA sample preparation kit (Illumina) and a Sciclone NGSx workstation (PerkinElmer). Library size distributions were validated using the Agilent 2200 TapeStation instrument (Agilent Technologies). Additional library quality control, blending of pooled indexed libraries, and cluster optimization were performed using a Life Technology-Invitrogen Qubit (ver2.0) fluorometer (Life Technologies-Invitrogen). RNA-seq libraries were pooled (duplex) and clustered onto a flow cell lane. Sequencing was performed using the Illumina HiSeq 2500 sequencer in rapid mode, employing a paired-end, 50-bp-read-length sequencing strategy. Image analysis and base calling were performed using Illumina's Real Time Analysis (ver1.18) software, followed by demultiplexing of indexed reads and generation of FASTQ files using Illumina's bcl2fastq conversion software (ver1.8.4).

Reads of low quality were filtered out prior to alignment to the MneHV7 custom reference genome sequence using the TopHat program (v2.0.12). Counts were generated from the alignments obtained with the TopHat program for each viral gene using the Python package HTSeq (v0.6.1; http://www-huber.embl.de/users/anders/HTSeq/doc/overview.html). Total viral read numbers were normalized by calculating the number of viral reads per million unique mapped genomic reads (VPMM) (25). The viral transcriptome expression profiles among the salivary gland specimens were analyzed using hierarchical cluster analysis (performed with CIMminer software). The Manhattan distance matrix was computed on the basis of the respective VPMM value calculated for each viral gene detected in each specimen. These values were used as input for hierarchical clustering using the complete linkage-clustering algorithm. Data sets representing the viral gene expression profiles were visualized by the use of heat maps generated with color-coded clustered image maps (CIMminer software) (26).

### Statistics.

Statistical analyses were performed using GraphPad Prism (ver6.01) software for Windows (GraphPad Software). To compare the overall read numbers from viral transcripts with the viral loads from whole-saliva or salivary gland tissue samples, two-tailed Spearman nonparametric correlation coefficients (*r*^2^ values) and the corresponding *P* values were calculated using 95% confidence intervals.

### Accession numbers.

The GenBank submission module (ver1.3.2) within the Geneious software package (ver6.1.6) was used to create a standard GenBank file for the MneHV7 genome and to submit the annotated sequence to GenBank. The sequence of the MneHV7 genome can be found under GenBank accession number KU351741. All the nucleotide and protein sequences analyzed in this report were derived from this annotated genome sequence. The complete RNA-seq data sets are available from the Gene Expression Omnibus (GEO) repository (GEO accession number GSE79944).

## RESULTS

### Selection of DNA samples for high-throughput sequencing.

To confirm that the MneHV7 DNA sequence segments that we previously detected in pigtailed macaque saliva by CODEHOP PCR indeed represented a novel macaque roseolovirus, we determined the genome sequence of MneHV7 by next-generation sequencing. We selected saliva as our source for viral DNA since we had previously determined that MneHV7 is highly prevalent in macaque saliva at consistently high levels (17). Thus, saliva samples from our study for which an adequate volume was available were screened by an MneHV7-specific qPCR to identify the samples with the highest viral loads. In order to enhance the recovery of viral DNA, whole-saliva samples were depleted of cellular material by centrifugation and filtration. Another source of genetic material contamination when extracting DNA from saliva is bacterial DNA. Thus, DNA samples with strong PCR signals for bacterial 16S rRNA sequences were eliminated.

Using these criteria, we selected and pooled three saliva samples collected at different times from the same naturally infected pigtailed macaque (macaque T05183). The final concentrated DNA sample contained 3.5 × 10^6^ copies of the MneHV7 genome per 100 ng of purified DNA. This sample was subsequently used to prepare a paired-end library that was sequenced on one lane of an Illumina HiSeq 2500 sequencer.

### Determination of MneHV7 genome sequence.

**FIG 1:**
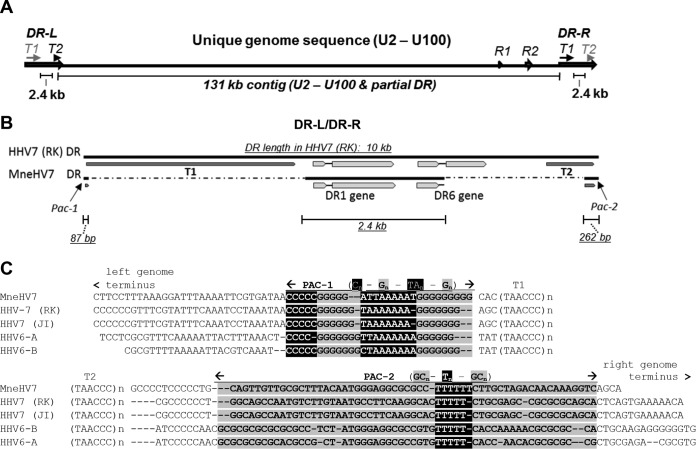
Schematic overview of MneHV7 genomic sequences obtained by *de novo* assembly. (A) A large contiguous sequence (contig) of 122 kb covering most of the unique MneHV7 genome segment and a smaller contig of 6.8 kb with similarity to the HHV-7 U95 and U100 genes were aligned to the HHV-7 (strain RK) reference genome. The gap between the two contigs consisted mostly of the R2 repeat region and was resolved by long-range PCR and Sanger sequencing. (B) An additional contig of 2.4 kb with similarity to HHV-7 end-terminal repeat elements (DR-L/DR-R) was also identified and contained the sequence for the DR1 gene and the first exon as well as most of the intron of the DR6 gene. (C) Sequences at both ends of the 122-kb contig extended into the end-terminal sequences and exhibited strong similarity with the conserved cleavage-packaging motifs, Pac-1 and Pac-2, of human roseoloviruses flanked by the telomeric repeat regions, T1 and T2. The segments containing conserved nucleotide reiterations (highlighted or shaded) for Pac-1 and Pac-2 and flanking telomeric repeat regions (T1, T2) are indicated. All sequences identified to be part of an end-terminal repeat element were duplicated at both genomic ends, on the basis of homology with HHV-7. The MneHV7 unique genome sequence and a segment of the DRs at the genomic termini, including the DR1 gene and the DR6 first exon, are available in GenBank under accession number KU351741.

Following high-throughput sequencing, a total of 215 million quality-filtered reads were mapped to the rhesus macaque genome in order to remove input reads originating from the macaque cellular genome. The remaining 36 million unmapped reads were then subjected to *de novo* assembly using the Velvet algorithm (22). The resulting contigs were searched for matches to the previously determined MneHV7 CODEHOP DNA polymerase amplicon sequence using blastn (27). A match was found, and the properties of the matching contig were used to further optimize the *de novo* assembly parameters to extend MneHV7 contig coverage. The divergence between the pigtailed and rhesus macaque genomic sequences, in addition to the presence of thousands of reads from mostly uncharacterized microorganism genomes, made the *de novo* assembly of MneHV7 reads challenging. Ultimately, this process generated a large contig of ∼122,000 bp covering a significant portion of the expected MneHV7 genome length (Fig. 1A). Another smaller contig of ∼6,800 bp with similarity to the sequences of the HHV-7 U95 and U100 genes was also identified and added to form a scaffold of the entire unique MneHV7 genome sequence with a mean read depth of 25 nucleotides. The gap between the two contigs, which mostly consisted of the R2 repeat region, was subsequently resolved by long-range PCR and Sanger sequencing. Sixteen additional very short unresolved sequences of 3 to 25 bp in length were mostly caused by the presence of single polynucleotide stretches and were resolved by mapping available overlapping reads to adjacent sequences using blastn, in addition to PCR amplification and subsequent Sanger sequencing. This process resulted in a sequence representing the entire unique genome region of MneHV7. Our final 130,851-bp MneHV7 draft genome included the entire unique genome sequence of MneHV7 as well as short sections of both ends of the end-terminal direct repeats (the right end of the left direct repeat [DR-L; 262 bp] and the left end of the right direct repeat [DR-R; 87 bp]) where the final contig extended beyond the unique genome region (Fig. 1A and B). Importantly, these partial end-terminal sequences had readily identified cleavage-packaging motifs (a Pac-1 motif on the left end and a Pac-2 motif on the right end of the unique genome region) flanked by several copies of the telomere-like TAACCC sequence motif that makes up the telomeric repeat regions T1 and T2 in human roseolovirus genomes (Fig. 1C) (28).

All roseoloviruses have a typical genome structure composed of a unique (U) genome region flanked by identical end-terminal DR regions of about 10 kb in length (DR-L–U–DR-R) oriented in tandem (15, 29, 30). HHV-7 DR regions contain open reading frames coding for the DR1 and DR6 genes. Further analysis of the contigs obtained following *de novo* assembly led to the identification of an additional short contig of 2.4 kb that showed similarity to HHV-7 end-terminal repeat elements (Fig. 1A and B). This contig contained the sequence for the entire DR1 gene and the first exon as well as most of the intron of the DR6 gene of MneHV7 (Fig. 1B). The complete DR6 sequence and the number of telomeric repeats present in the T1 and T2 regions at each end of the MneHV7 genome remain to be determined.

For the final MneHV7 genome assembly, all available sequences belonging to the end-terminal DR region were duplicated at each end of the MneHV7 genome on the basis of alignments with the HHV-7 genome. The resulting MneHV7 genome sequence is available in GenBank under accession number KU351741.

### MneHV7 genome annotation.

**FIG 2:**
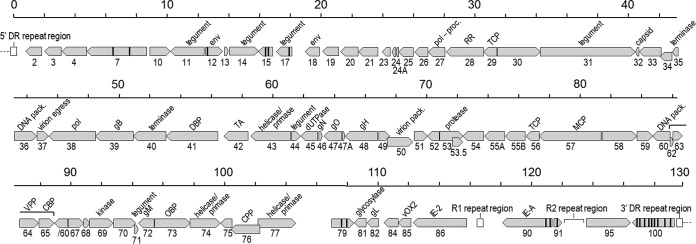
Map of the ORFs identified in the MneHV7 genome. The genomic positions (130-kb unique genome sequence, excluding DRs), lengths, and transcription directions of 84 MneHV7 ORFs (in gray) were identified on the basis of similarity with the annotated HHV-7 RK strain genome using GATU software. ORF boundaries were determined on the basis of the position of start and stop codons and, when applicable, confirmed by the presence of appropriately located poly(A) signals and TATA boxes. Vertical black lines within an ORF indicate predicted splice sites, while the longer-ranged splice between the two protein-coding exons in U60 is indicated with a bar. Internal repeat regions R1 and R2 and the end-terminal direct repeat regions (DR-L [5′], DR-R [3′]) are represented by white rectangles. proc., processing; pack., packaging; RR, ribonucleotide reductase; TCP, triplex capsid protein; DBP, single-stranded DNA-binding protein; MCP, major capsid protein; CPP, capsid portal protein; TA, transactivator.

Annotation of the MneHV7 genome sequence was performed using the Genome Annotation Transfer Utility (GATU) (23) and the HHV-7 RK strain sequence as the reference genome. Open reading frames with similarity to all 84 known HHV-7 ORFs spanning the entire genomic region between the U2 and U100 genes were identified (Fig. 2). The MneHV7 ORFs identified, including their direction, size, and percent amino acid sequence identity to the corresponding HHV-7 ORFs, are listed in Table 1. Similar to HHV-7, the MneHV7 sequence contained a homolog of the HHV-7-specific U55B gene, whereas it lacked sequences similar to the HHV-6 unique genes, U22, U83, and U94. These observations confirmed the previous status of MneHV7 as a new HHV-7 homolog.

**TABLE 1:** ORFs predicted from MneHV7 genome sequence with number of exons, coding direction, protein length, and sequence homology to the corresponding ORFs in HHV-7 RK

ORF	No. of exons	Direction	Length (no. of aa)^*a*^		% aa sequence identity^*b*^	Function or homolog
			HHV-7 RK	MneHV7		
DR1	2	Forward	507	503	47.8	Unknown
DR6	Exon 1^*c*^	Forward	139	109	56.0	Unknown
U2	1	Reverse	359	352	61.8	Tegument protein
U3	1	Reverse	384	388	67.5	Tegument protein
U4	1	Reverse	542	542	60.9	Unknown
U7	3	Reverse	1,232	1,228	74.8	Unknown
U10	1	Forward	451	448	66.4	Unknown
U11	1	Reverse	755	734	45.1	Major tegument protein/capsid binding
U12	2	Forward	333	331	68.3	Envelope glycoprotein/intracellular signaling
U13	1	Forward	98	95	50.0	Unknown
U14	1	Forward	648	630	66.9	Tegument protein
U15	3	Reverse	191	190	92.7	Glycoprotein
U17	2	Reverse	330	325	72.3	Viral ICA tegument protein/apoptosis
U18	1	Reverse	295	299	67.9	Envelope glycoprotein/apoptosis
U19	1	Reverse	325	344	57.1	Apoptosis
U20	1	Reverse	391	381	32.2	Membrane protein
U21	1	Reverse	430	428	53.7	Membrane protein
U23	1	Reverse	171	171	31.2	Membrane protein
U24	1	Reverse	82	83	47.0	Unknown
U24A	1	Reverse	56	54	53.7	Unknown
U25	1	Reverse	320	319	59.6	Tegument protein
U26	1	Reverse	293	295	54.9	Envelope protein/possibly membrane fusion
U27	1	Reverse	364	364	84.1	DNA polymerase processivity subunit
U28	1	Reverse	806	801	64.1	Ribonucleotide reductase subunit
U29	1	Reverse	286	284	69.9	Triplex capsid protein/capsid morphogenesis
U30	1	Forward	938	937	63.2	Tegument protein/virion morphogenesis
U31	1	Forward	2,059	2,056	62.3	Large tegument protein/capsid transport
U32	1	Reverse	90	88	79.5	Capsid protein/capsid morphogenesis
U33	1	Reverse	477	471	67.4	Unknown
U34	1	Reverse	258	258	71.3	Nuclear egress membrane protein
U35	1	Reverse	104	104	74.0	DNA-packaging protein/DNA encapsidation
U36	1	Forward	485	483	70.1	DNA-packaging protein/DNA encapsidation
U37	1	Forward	259	258	76.4	Nuclear egress lamina protein
U38	1	Reverse	1,013	1,012	77.3	DNA polymerase catalytic subunit (pol)
U39	1	Reverse	822	817	74.0	gB/cell entry, cell-cell spread
U40	1	Reverse	721	716	68.2	DNA-packaging terminase/DNA encapsidation
U41	1	Reverse	1,131	1,131	82.9	Single-stranded DNA-binding protein/DNA replication
U42	1	Reverse	516	517	76.6	RNA-binding protein/RNA regulation
U43	1	Reverse	861	860	72.8	Helicase/primase complex protein
U44	1	Forward	203	203	65.5	Tegument protein/virion morphogenesis
U45	1	Reverse	379	367	60.2	Deoxyuridine triphosphatase
U46	1	Forward	86	81	60.0	Glycoprotein N (gN)/virion morphogenesis
U47	1	Reverse	313	382	40.2	Glycoprotein O (gO)/virion morphogenesis
U47A	1	Forward	56	56	54.5	Envelope glycoprotein 24
U48	1	Reverse	690	691	68.5	Glycoprotein H (gH)/cell entry, cell-cell spread
U49	1	Forward	239	230	68.3	Nuclear protein
U50	1	Forward	554	553	68.3	Virion packaging protein
U51	1	Forward	294	291	49.3	Envelope protein/possibly intracellular signaling
U52	1	Reverse	254	255	68.4	Unknown
U53	1	Forward	513	500	63.4	Capsid maturation protease
U53.5	1	Forward	230	226	63.9	Capsid scaffold protein
U54	1	Reverse	454	454	71.1	Tegument protein pp65/immune regulation
U55A	1	Reverse	427	427	49.9	DNA regulation
U55B	1	Reverse	430	429	50.9	Unknown
U56	1	Reverse	293	293	80.5	Triplex capsid protein/capsid morphogenesis
U57	1	Reverse	1,345	1,345	80.0	Major capsid protein/capsid morphogenesis
U58	1	Forward	775	763	72.1	Unknown
U59	1	Forward	347	343	49.4	Tegument protein
U60	2	Reverse	663	663	80.4	DNA-packaging terminase subunit
U62	1	Forward	75	72	62.5	Unknown
U63	1	Forward	211	207	77.7	Unknown
U64	1	Forward	439	439	56.5	Virion-packaging protein
U65	1	Forward	330	329	74.2	Capsid binding protein
U67	1	Forward	346	346	69.9	Unknown
U68	1	Forward	114	114	57.0	Tegument protein/virion morphogenesis
U69	1	Forward	546	522	67.6	Serine/threonine protein kinase
U70	1	Forward	480	480	71.0	DNase/DNA processing
U71	1	Forward	73	78	63.0	Tegument protein
U72	1	Reverse	346	345	83.5	Glycoprotein M (gM)/virion morphogenesis
U73	1	Forward	787	788	69.8	OBP/DNA replication
U74	1	Forward	659	656	53.7	Helicase-primase subunit/DNA replication
U75	1	Reverse	256	250	54.6	Tegument protein/virion morphogenesis
U76	1	Reverse	640	638	73.3	Capsid portal protein
U77	1	Forward	820	820	86.2	Helicase/primase complex protein
U79	3	Forward	506	438	51.7	Gene regulation/DNA replication
U81	1	Reverse	254	252	74.6	Uracil-DNA glycosylase/DNA repair
U82	1	Reverse	246	250	66.8	Glycoprotein L (gL)/cell entry, cell-cell spread
U84	1	Reverse	310	308	49.7	Unknown
U85	1	Reverse	280	277	56.5	ox-2 homologue (viral OX-2)/immune regulation
U86	1	Reverse	1,205	1,157	55.8	Regulatory protein IE-2/gene regulation
U90	3	Reverse	1,199	1,008	46.0	Immediate early protein IE-A/gene regulation
U91	2	Forward	153	148	53.8	Membrane protein
U95	1	Forward	940	951	48.3	Unknown
U100	10	Reverse	603	599	56.1	Glycoprotein Q (gQ)/cell entry

a HHV-7 RK, HHV-7 RK strain genome (GenBank accession number NC_001716); MneHV7, MneHV7 genome (GenBank accession number KU351741, this study); aa, amino acids.

b Percent amino acid (aa) sequence identity to the corresponding ORFs from the HHV-7 genome (RK strain).

c A 2.4-kb contig contained the sequence for both DR1 gene contigs but only for the first exon of the DR6 gene, including some intergenic region.

The positions of ATG start codons, stop codons, and similarity with the HHV-7 (RK strain) annotation were used to predict open reading frames. The presence and locations of TATA box consensus sequences as well as consensus polyadenylation signal sequences were identified. MneHV7 proteins with the highest percent amino acid sequence similarity to HHV-7 proteins (>90%) were U27 (DNA polymerase processivity factor), U35 (DNA packaging), U41 (single-stranded DNA-binding protein), U56 (triplex capsid protein), U57 (major capsid protein), U72 (envelope glycoprotein M), and U77 (helicase/primase complex protein). Twenty-seven additional proteins were 80 to 90% similar, 18 proteins were 70 to 80% similar, and 38 proteins shared <70% amino acid sequence similarity (Table 1). For MneHV7 homologs of known HHV-7 spliced genes, all splice donor and acceptor consensus sequences at intron-exon boundaries were determined on the basis of positional similarity to HHV-7 and by inspection of the MneHV7 sequence for such splice donor and acceptor consensus sequences. Intron-exon boundaries were adjusted as necessary (Table 2). The sizes and positions of the predicted introns were highly conserved between MneHV7 and the HHV-7 RK strain annotation, as was the presence of conserved splice junction sequence elements (30).

**TABLE 2:** Predicted splice donor and acceptor sites in MneHV7 genome

ORF	Exon no.	Sequence^*a*^			
		HHV-7 RK acceptor	HHV-7 RK donor	MneHV7 acceptor	MneHV7 donor
Consensus		YYYYYYYYYYYNY**AG**G	MAG**GT**RAGT	YYYYYYYYYYYNY**AG**G	MAG**GT**RAGT
DR1	1	TCTCTTCTATCAC**AG**A	CAT**GT**AAGC	GTCTTCTCGTCGC**AG**T	CCG**GT**ACTC
	2	TTTGCTCTATCGC**AG**G	None	TTTCGCCTCTCGC**AG**A	None
DR6	1	CTCCGCGCTGTTC**AG**C^*b*^	GCG**GT**GAGT	GTCTCTCTGTCAC**AG**C	GCG**GT**GAGT
	2	TCTACATCCCGGC**AG**C	None	Not determined	Not determined
U7	1	None	TAA**GT**AAGT	None	TGA**GT**AAGT
	2	AAGTTAATTTTGC**AG**A	TAG**GT**ATGT	ACCTTTTTTTTTT**AG**A	CAG**GT**ACAT
	3	GATGTTCTTTTTC**AG**A	None	TGTTTTTTTTAAT**AG**G	None
U12	1	TTTCTCTTTGATT**AG**A^*b*^	CTG**GT**ATGA	TTTTTCTTTAACT**AG**A	TGG**GT**AAGA
	2	AACTTTTTTTCAC**AG**C	None	TCATATTTCTTAT**AG**C	None
U15	1	ACTCTTCTTTGTG**AG**A^*b*^	ACG**GT**GAGT	None	ACG**GT**AGGT
	2	CTCTTTTATTTTC**AG**G	GCG**GT**AAGA	TGTTTTTTATTTC**AG**A	GCG**GT**AAGA
	3	TTTTTTTTTCTTT**AG**T	None	TTGTTTTTTTTTC**AG**T	None
U17	1	None	TAT**GT**AAGT	None	TAT**GT**AAGA
	2	TTGTTGTTTTCAT**AG**G	None	TGTTTTTTTTTTT**AG**G	None
U60	1	None	CAC**GT**AAGT	None	CAC**GT**AAGT
	2	TCATTTTCTTCTC**AG**A	None	CATTTTCTTTCCT**AG**A	None
U97	1	None	AAG**GT**TAGT	None	AAG**GT**AAAT
	2	AACATGTTTTCTT**AG**A	CAG**GT**GGGT	TTTTTTATGTTTC**AG**A	CAG**GT**GGGT
	3	GTTTCTTTCTTTT**AG**G	None	TTTCTTTTTTTTT**AG**G	None
U90	1	GGTTTGTTATTGT**AG**G	TGA**GT**AGGT	GGCTTGTTTTTGT**AG**G	CGA**GT**ATGT
	2	TAAATTTTATTAC**AA**G	CAG**GT**ATTT	AAATCTTTATTGT**AG**A	CAG**GT**AATA
	3	TTCTTTAAATTCT**AG**C	None	TCTTTTAAATCCT**AG**C	None
U91	1	None	CAG**GT**TTGT	None	CAG**GT**TAGT
	2	TATTTTTTCTTGT**AG**A	None	TTATTTTTCTTGT**AG**A	None
U100	1	AAAATCTCTTCGC**AG**A	ACA**GT**AAGT	AAAAATCTTTTGC**AG**G	ACA**GT**AAGT
	2	TTTAATTCTTCTA**AG**G	ATG**GT**AAGC	TTTTTTTTAAACC**AG**G	CTG**GT**AAGA
	3	GTACCCGCTTATT**AG**T	AGT**GT**AAGT	CTTTTCTCAAACC**AG**C	AGT**GT**TTGT
	4	TATTTTTTTTTTT**AG**A	AAT**GT**AAGA	TTCTTTTTTTTTT**AG**T	CAT**GT**GAGT
	5	AATTGTGTTTCGC**AG**T	CAG**GT**AAAT	CCCTTTTCTCTTA**AG**C	AAG**GT**AAAT
	6	GCTTCTTCATCCT**AG**A	TTG**GT**AATT	TTTTTTAATCCGC**AG**T	GTG**GT**ATGT
	7	TTTTTTTCATACC**AG**C	ACA**GT**GGAA	TTTTTATCTCTAC**AG**C	AAA**GT**GGAA
	8	TTTTTTTAATTCT**AG**C	CAT**GT**GAGT	TAACTGCGACCAA**AG**A	TGT**GT**AAGT
	9	ATTTCTCGTTCGC**AG**C	CAG**GT**GAGC	AAATGTCTTTCTT**AG**G	CAG**GT**AAAA
	10	CATTTTCTCTTTT**AG**T	None	TTTATTTTTTTTT**AG**C	None

a None, no conserved splice acceptor or donor site was found; not determined, no DR6 exon 2 sequence with which to determine the presence of conserved splice sites was available; boldface indicates conserved nucleotides.

b In the HHV-7 RK genome, new consensus splice acceptor sites were found near the start codons of the first exons of DR6, U12, and U15. Splice acceptor sites near the start of first exons indicate the possible existence of an upstream noncoding exon.

We catalogued all polymorphic nucleotide positions where two variant nucleotide sequences were observed and where the number of reads containing one of the nucleotides represented at least 25% of all reads at that particular position (data not shown). The nucleotide occurring in the majority of reads was chosen for the MneHV7 consensus genome sequence. Of 77 variant positions located in predicted ORFs, 25 led to amino acid changes, but only 15 of those were nonconservative changes. However, additional sequencing information from viral DNA isolated from different macaques will be required to evaluate whether these variant nucleotide positions (single nucleotide polymorphisms) are truly present or resulted from polymerase errors.

### Phylogenetic analysis.

**FIG 3:**
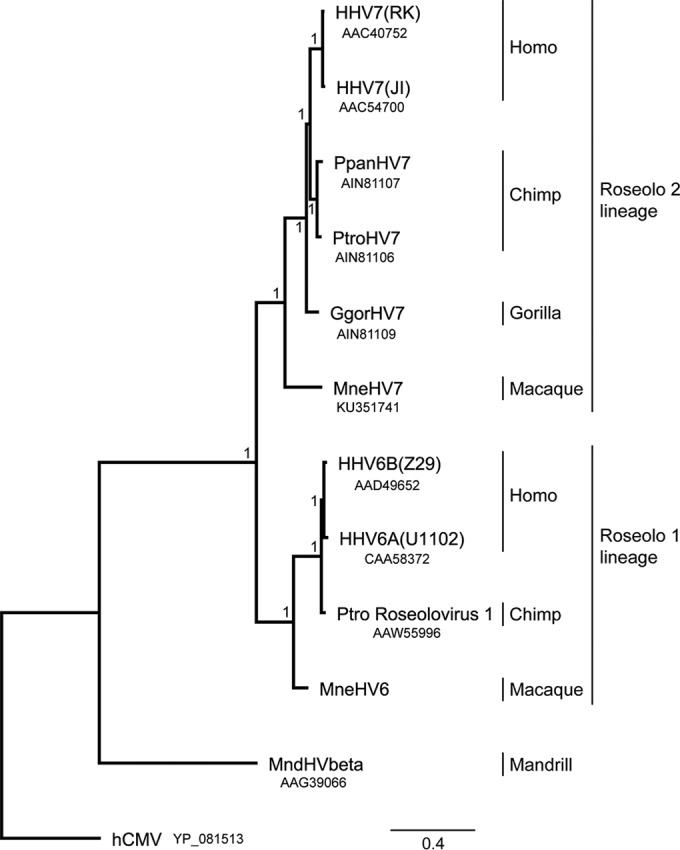
Phylogenetic relationship of MneHV7 with members of the roseolovirus subfamily. The DNA polymerase (DNA pol) amino acid sequences available from 12 human and nonhuman primate roseoloviruses, including MneHV7, were compared using maximum likelihood analysis. GenBank accession numbers are provided beneath the virus designations. The hCMV/HHV5 DNA pol sequence was used as an outgroup. All resolved nodes have posterior probabilities of 1 (chi-square values) and are thus well supported. The scale bar indicates the amino acid sequence divergence among sequences; branch lengths reflect the relative relatedness of the sequence. Known members of the Roseolovirus genus from both provisional lineages (roseolo 1 lineage and roseolo 2 lineage) are shown. HHV, human herpesvirus; Ppan, Pan paniscus or P. bonobo; Ptro, Pan troglodytes (common chimpanzee); Ggor, Gorilla gorilla (lowland gorilla); Mnd, mandrill.

Recently, Lavergne et al. have reported the existence of HHV-7 simian homologs naturally infecting African great apes, including chimpanzees and gorillas (18). The study determined the complete sequence of the viral polymerase gene and a partial sequence of the glycoprotein B gene for each host species. We constructed a phylogenetic tree representing the evolutionary relationships between all roseoloviruses known to date using the corresponding sequence from MneHV7, with the hCMV sequence used as an anchor. The tree was based on a MUSCLE alignment (31) of all known roseolovirus DNA polymerase amino acid sequences, followed by maximum likelihood (PhyML) analysis (24). As shown in Fig. 3, the MneHV7 sequence clustered closely with the sequences of the simian HHV-7 homologs and those of two HHV-7 strains (RK and JI) in a lineage (provisionally named the roseolo 2 lineage) separate from the lineage containing both human HHV-6A and HHV-6B species and HHV-6 primate homologs (provisionally named the roseolo 1 lineage) (17). The clustering relationship between HHV-7 and its homologs in macaques and great apes is consistent with the long-term coevolution of roseoloviruses with their respective primate host species.

### The major MneHV7 internal repeat elements, R1 and R2.

**FIG 4:**
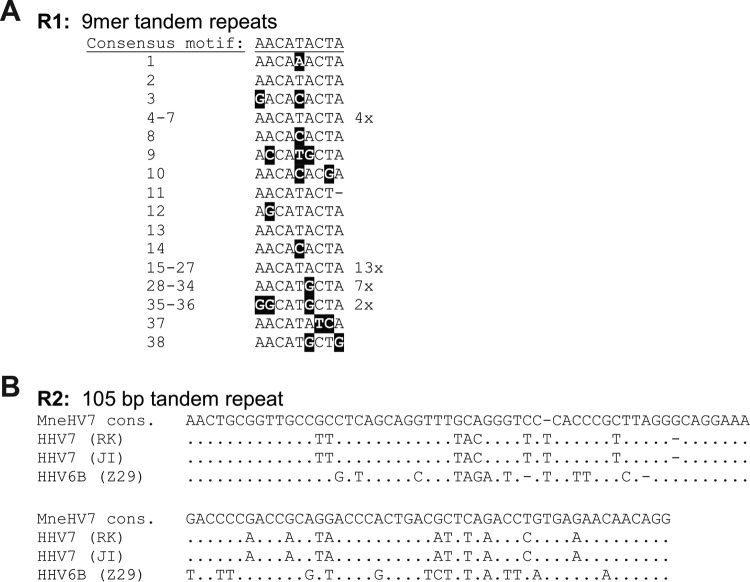
Nucleotide sequences of MneHV7 repeat regions R1 and R2. (A) The 9-bp MneHV7 R1 repeats (R1) are shown in the order in which they appear in the sequence, with nucleotides diverging from the consensus motif being highlighted in black. The rate of occurrence of identical motifs repeated multiple times is indicated to the right of the respective motif. (B) The 105-bp MneHV7 R2 consensus (cons.) repeat motif (R2) was aligned with the R2 motifs of HHV-7 (strains RK and JI) and HHV-6B (strain Z29). Identical residues are indicated by dots.

Genome analysis showed that, similarly to the HHV-7 genome, the MneHV7 unique genomic sequence contained two internal repeat regions, R1 and R2 (15). As in the HHV-7 genome, the MneHV7 R1 repeat region is located between the adjacent ORFs U86 and U89. The sequence for the MneHV7 R1 repeat region was part of the initial large contig obtained by *de novo* assembly and was also confirmed by PCR amplification and Sanger sequencing. The finalized MneHV7 R1 region sequence is composed of 38 copies of a 9-nucleotide-long conserved sequence motif with only minor variations between repeats (Fig. 4A). The sequence, length, and number of these reiterated motifs were markedly different from those of both the HHV-7 JI and RK strain R1 regions, which are composed of much longer direct and inverted repeats (15). However, we found that the regions flanking MneHV7 R1 are comprised of multiple copies of the sequence TAAAT, as is the case for HHV-7.

The MneHV7 R2 repeat region is located between ORFs U91 and U95. The complete sequence of the MneHV7 R2 repeat region was determined using specific long-range PCR amplification with multiple primer pairs and Sanger sequencing. The number of repeats was evaluated both by calculating the respective amplicon lengths from polyacrylamide gels and by mapping the available reads to the R2 sequence at the same read depth used for the surrounding sequence. We determined that the MneHV7 R2 region is composed of approximately 20 conserved copies of a 105-bp-long motif, plus one final truncated copy containing the first 75 nucleotides of the motif (Fig. 4B). The majority of the repeats shared an identical nucleotide sequence. Each of only five repeats varied by a single nucleotide from the consensus sequence, while the last truncated motif contained three nucleotide variations. In contrast to the R1 region, the consensus R2 repeat motif in the MneHV7 genome sequence shared significant sequence similarity with the HHV-7 R2 consensus motif (78 to 82% with both strains RK and JI), was identical in length to the HHV-7 R2 consensus motif, and contained numbers of repeats similar to the numbers in the HHV-7 R2 consensus motif.

### Conservation of the MneHV7 U73-encoded OBP and the OBP-binding region in the origin of lytic replication.

**FIG 5:**
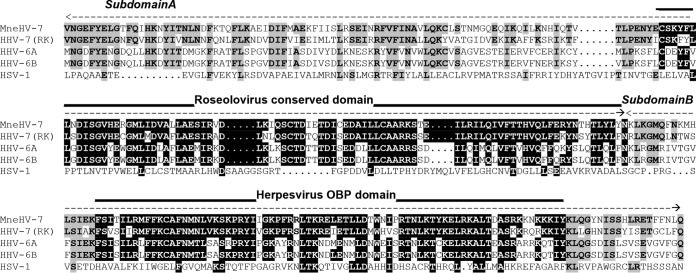
Comparison of the DNA-binding domains in the OBP of MneHV7 and different human herpesviruses. Alignment of the carboxy-terminal amino acid sequences of OBPs from MneHV7 and different human herpesviruses, including the human roseoloviruses HHV-7 (GenBank accession number AAC40752), HHV-6A (GenBank accession number CAA58372), and HHV-6B (GenBank accession number AAD49652) and HSV-1 (GenBank accession number X14112), is shown. The boundaries of subdomains A and B are indicated by dashed lines. Conserved amino acids among highly conserved regions (the roseolovirus conserved domain and the herpesvirus OBP domain) within subdomains A and B are highlighted. Amino acid conservation in other positions is shaded gray.

Human roseoloviruses rely on the U73-encoded origin-binding protein (OBP) to initiate the process of DNA replication. OBP interacts with two specific sites, OBP-1 and OBP-2, within the origin of lytic replication (oriLyt), which is positioned in the intergenic region between ORFs U41 and U42 (13, 32). Two amino acid domains located at the carboxy terminus of OBP were previously identified as subdomains A and B and have been shown to interact with OBP-1 and OBP-2, respectively (14). To assess the degree of sequence conservation within these two subdomains, we aligned the carboxy-terminal sequence of MneHV7 U73 with OBP sequences from HHV-7, HHV-6A, and HHV-6B. As shown in Fig. 5, a segment termed the roseolovirus conserved domain of subdomain A is highly conserved among roseoloviruses (80% amino acid sequence identity between subdomains A of MneHV7 and HHV-7; 64% and 65% identity between subdomains A of MneHV7 and HHV-6A and -B, respectively). In comparison, the same sequence alignment between MneHV7 and herpes simplex virus 1 (HSV-1) shows only 7% identity. Another highly conserved region, the herpesvirus OBP domain, was identified in subdomain B (80% amino acid identity between subdomains B of MneHV7 and HHV-7; 67% and 70% identity between subdomains B of MneHV7 and HHV-6A and -B, respectively). This 76-amino-acid long region has been reported to be conserved among several herpesvirus OBPs. There is 20% identity between the MneHV7 and HSV-1 amino acid sequences from the herpesvirus OBP domain.

In agreement with previous observations, subdomain B is more highly conserved at the amino acid level than subdomain A (75% identity for subdomain B versus 62% identity for subdomain A between MneHV7 and HHV-7; for subdomain B, 62% and 64% identity between MneHV7 and HHV-6A and -B, respectively; for subdomain A, 50% and 51% identity between MneHV7 and HHV-6A and -B, respectively).

**FIG 6:**
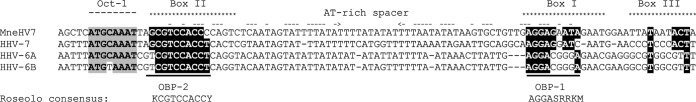
Comparison of OBP-binding sites in the oriLyt regions of MneHV7 and human roseoloviruses. The alignment of the OBP-binding domains within the oriLyt regions of the human roseoloviruses and the predicted oriLyt region of the MneHV7 sequence is shown. Conserved nucleotides within box I (OBP-1) and box II (OBP-2) and possible box III binding sites are highlighted, and the roseolovirus consensus sequences for OBP-1 and OBP-2 are indicated below the alignment. The conserved residues of the Oct-1 transcription factor binding site downstream of box II are shaded in gray. The nucleotides contributing to the dyad symmetry across box I, box II, and the AT-rich spacer sequence in the MneHV7 genome are indicated by dashed lines above the MneHV7 sequence.

We also compared the nucleotide sequences of the OBP-binding sites, OBP-1 and OBP-2, within the MneHV7 putative oriLyt region (13). As shown in Fig. 6, the sequences of the MneHV7 OBP-binding site regions are highly similar to the conserved OBP-binding site sequences in different HHV-7 strains for both OBP-1 (Fig. 6, box I; 7 out of 9 bp are conserved) and OBP-2 (Fig. 6, box II; 9 out of 10 bp are conserved). The similarities extended to the presence of sequences with dyad symmetry between the two OBP-binding sites flanking an AT-rich spacer element. The two segments of DNA whose base pair sequences are inverted repeats of each other are delineated (converging dashed lines in Fig. 6). In addition, an Oct-1 binding site upstream of OBP-2 is fully conserved between HHV-7 and MneHV7. In contrast, a previously identified third binding site (box III) found in herpes simplex viruses (HSV-1 and HSV-2) and in different HHV-7 strains, but not in HHV-6B and HHV-6A, was not conserved in MneHV7.

### Viral mRNA expression profile in salivary gland tissues naturally infected with MneHV7.

Little is known about the HHV-7 transcriptome in infected cells. A few viral transcripts have been detected in RNA isolated from cultures of a productively infected T cell line and have been described (16). Previous attempts to detect HHV-7 transcripts in RNA isolated from PBMCs with detectable HHV-7 DNA have failed (16). Similar to HHV-7, MneHV7 DNA is only occasionally detected by specific qPCR in macaque PBMCs and is detected at low levels (17). Consequently, we chose to characterize MneHV7 RNA transcripts in macaque salivary gland tissues, where MneHV7 DNA was consistently detected by qPCR in our previous study (17).

To gain insight into MneHV7 gene expression patterns in naturally infected tissues, we used high-throughput RNA sequencing (RNA-seq) on poly(A)-selected RNA obtained from parotid gland specimens from nine infected macaques. As we reported before, these animals were part of a group of 12 pigtailed macaques from a previously described simian immunodeficiency virus (SIV) vaccination study (20). Of the nine animals, three (macaques M-3126, M-3182, and K-3258) were classified as long-term nonprogressors, with only low levels of the SIV/human immunodeficiency virus hybrid (SHIV) consistently being detected in plasma. The remaining six animals (macaques L02393, M02383, M-2298, M-5226, T-5183, and M-3240) had developed a sterilizing immunity with no detectable SHIV in plasma following challenge. All tissues had been snap-frozen shortly after necropsy and kept stored at −80°C. The 50-bp-long RNA-seq paired reads obtained for each animal were separately aligned to the MneHV7 genome using the TopHat2 aligner (33), and reads aligned to individual viral genes were enumerated using the HTSeq software package (34).

First, we examined the overall viral read number in each salivary gland specimen. Low numbers of viral reads aligned to the MneHV7 genome. To allow comparison between data sets, HTSeq data were normalized by calculating the number of viral reads per million unique mapped genomic reads (VPMM) (35). While expression of the data as a function of gene length (e.g., as the number of reads per kilobase of exon per million mapped reads) might strengthen some of the relationships between expressed genes, normalization of the data as VPMM was better suited to our particular data set because of the relatively low read coverage. The VPMM values based on the overall viral read number ranged from 7.2 (macaque T-5183) to 0.1 (macaque M-2383). The low number of viral reads is likely due to the fact that we used tissue specimens from naturally infected macaques. Not all cell types present in salivary gland tissue are expected to be equally susceptible to MneHV7 infection and able to support viral replication. Moreover, the salivary glands studied did not present any pathological symptoms, suggesting that MneHV7 infection in these animals was under cellular control. This is in contrast to the findings of other viral transcriptome studies, where tissues with pathological conditions, often tumors, have been used and for which higher numbers of viral reads have been reported (25, 36).

**FIG 7:**
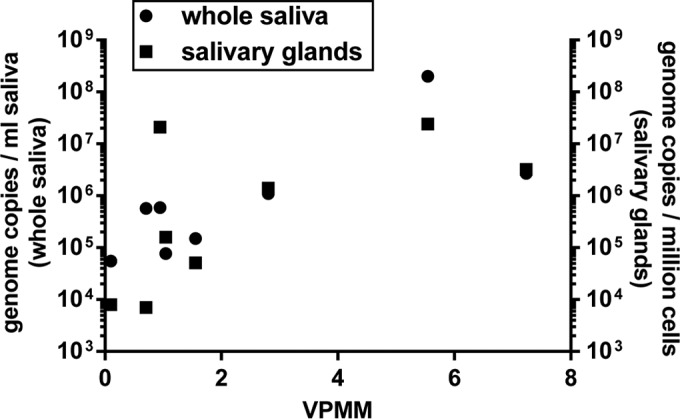
Correlation between MneHV7 viral loads in saliva or salivary glands and viral transcript expression in salivary gland tissues. The MneHV7 viral loads in whole saliva or salivary gland tissues were compared to the total number of VPMM in the corresponding salivary gland tissues. The statistical significance of the correlations was assessed from two-tailed Spearman nonparametric correlations with 95% confidence intervals (for whole saliva, *r*^2^ = 0.80 and *P* = 0.014; for salivary glands, *r*^2^ = 0.70 and *P* = 0.040).

Previously, we observed a strong correlation between MneHV7 viral loads in orogastric tissues (salivary glands and pyloric stomach) and whole saliva for all the macaques in our study (17). Hence, we examined the relationship between the normalized overall levels of viral gene expression seen in our RNA-seq data sets and the MneHV7 viral loads measured by qPCR in the corresponding salivary glands and whole-saliva samples. Statistically significant correlations between the normalized read number mapping to the MneHV7 genome (VPMM) and both the MneHV7 viral loads in saliva (*r*^2^ = 0.80, *P* = 0.014) and the MneHV7 viral loads in salivary gland tissues (*r*^2^ = 0.70, *P* = 0.040) were detected for all nine data sets (Fig. 7). These data indicate that the level of MneHV7 gene expression in salivary glands correlates with the viral load not only in the salivary gland tissue but also in whole saliva.

**FIG 8:**
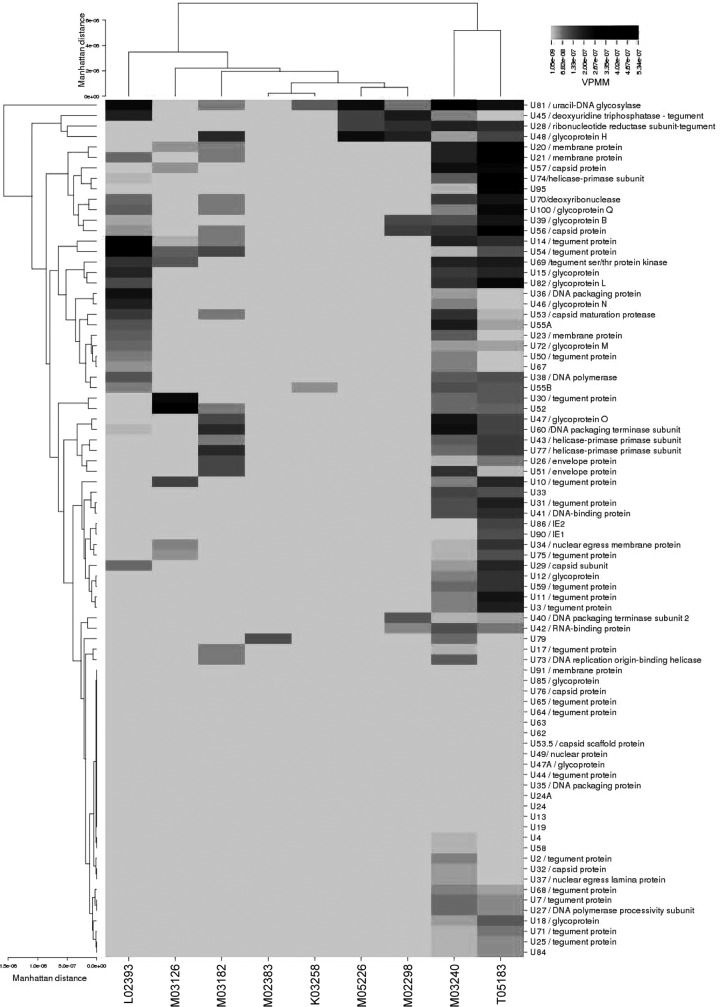
Hierarchical cluster analysis of MneHV7 mRNA expression profiles in nine macaque salivary gland tissues. The heat map shows the number of VPMM for each MneHV7 gene in salivary gland tissue from nine different pigtailed macaques. Color intensity represents the number of VPMM for each identified viral gene across all tissue samples. The Manhattan distance matrix was used as the input for generating the dendrograms using the complete linkage-clustering algorithm.

Reads mapping to all 84 viral ORFs identified within the MneHV7 genome were enumerated using HTSeq analysis. The range of the normalized read numbers mapped to each MneHV7 gene in the different macaque salivary gland specimens is shown in the form of a heat map (Fig. 8). The expression profile of transcripts associated with MneHV7 varied between specimens, indicating the heterogeneity of viral gene expression between individual hosts even within a specific type of tissue. The low overall number of viral reads prevented us from accurately determining the relative abundance of each transcript. Nonetheless, our characterization of the MneHV7 transcriptome in naturally infected salivary glands provides a first comprehensive list of viral proteins of biological interest for which quantitative expression could be further investigated using specific RT-qPCR.

To assess global differences in the MneHV7 gene expression profiles among infected salivary gland samples, we performed hierarchical clustering analysis using CIMminer software (26). Hierarchically clustered viral genes (rows) and salivary gland tissue from each macaque (columns) are shown in dendrograms in Fig. 8. The viral gene clustering is the result of a mathematical prediction of the putative relationship between the expression of different viral genes and may indicate an interaction or synergism between genes within a particular cluster. The viral gene expression profiles found in salivary glands from two animals, macaques M03240 and T-5183, clustered together, showing transcripts from a large number of different viral genes. Reads mapping to transcripts from early and late genes included the viral polymerase (U38) as well as several tegument proteins (U3, U7, U10, U1, U14, U25, U30, U31, U54, U59, U68, U71, and U75) and glycoproteins (U12, U15, U18, U39/gB, U47/gO, U48/gH, U72/gM, U82/gL, U100/gQ). These findings are consistent with active MneHV7 replication in salivary glands from these two macaques at the time of necropsy. Overall, the MneHV7 gene expression profiles of the remaining seven animals clustered separately from those of macaques M-3240 and T-5183. While the individual profiles formed a loose cluster, they were quite different. Within this group, the profiles for macaques M-2383 and K-3258 clustered together, with only one and two viral transcripts being detected (U79 and U55B in macaque M-2383 and U81 in macaque K-3258). The expression profiles for macaques M02298 and M-5226 also clustered together, with very few viral transcripts being detected. Both macaques shared the expression of viral transcripts from U28, U45, U48/gH, and U81. The viral transcriptomes of the remaining three animals (macaques L02393, M03126, and M03182) showed the transcription of 10 to 25 viral genes. The viral gene expression profiles associated with salivary glands from the three SHIV-positive macaques (macaques M03126, M03182, and K03258) did not cluster separately from those associated with salivary glands from SHIV-negative macaques, indicating that SHIV infection had no significant impact on global MneHV7 gene expression.

The MneHV7 gene expression dendrogram (the rows in Fig. 8) illustrates the associations between viral genes expressed in naturally infected macaque salivary glands. U81, a uracil-DNA glycosylase, was the viral gene that was the most frequently detected among the different salivary glands. It clustered closely with other genes, U28 and U45, which, like U81, are also involved in DNA repair and nucleotide metabolism, and with the viral glycoprotein gH. This particular group of genes was detected both in the two animals with a broad range of viral gene expression (macaques M03240 and T05183) and in two other animals with much more limited gene expression (macaques M02298 and M05226). In contrast, another distinct cluster of genes composed of U7, U18, U25, U27, U68, U71, and U84 was detected only in the two macaques with the highest number of viral transcripts.

Viral glycoproteins gQ and gB grouped in a cluster separate from the glycoproteins gL, gN, and gM, which grouped together with DNA polymerase U38 and several viral structural proteins (U14, U15, U23, U50, and U54). Glycoprotein O is part of yet another gene cluster that also contains U43 and U77, which are involved in DNA replication during productive infection. Both immediate early transactivators, U86 and U90 (coding for IE-1 and IE-2, respectively), clustered together. Finally, no transcript could be detected for a number of MneHV7 genes in any salivary gland tissue section. This is similar to what has been observed for Epstein-Barr virus expression profiles in a cohort of non-Hodgkin's diffuse large B cell lymphomas (25).

These data suggest that salivary glands from different animals were infected with MneHV7 at different stages of its life cycle. Since our viral transcriptome data were obtained from mRNA from total salivary gland tissues, it is possible that the different viral gene expression patterns observed reflect infection of different cell types at different stages in the viral life cycle.

### Detection of MneHV7 protein in macaque salivary glands.

Salivary glands are a major site for HHV-7 persistence and active replication (9, 37, 38). We have previously reported that MneHV7 is consistently detected in saliva and in macaque salivary gland tissues, similar to what is observed in humans (17). Our previous biological characterization of MneHV7 natural infection in pigtailed macaques relied on MneHV7-specific qPCR for detection and quantification of the viral genomes. To further characterize MneHV7 infection, we assessed the expression of viral protein in macaque salivary gland tissue by immunohistochemistry (IHC). IHC staining for the presence of viral protein in tissue sections allowed us not only to visualize infected cells but also to identify infected cell types.

Two HHV-7-specific monoclonal antibodies, clones 5E1 and KR4, have previously been used for staining of HHV-7 protein in infected human salivary gland tissues by IHC (9, 35, 39). Both were reported to produce similar staining patterns. Since the 5E1 antibody was no longer available, we obtained the KR4 antibody, which is thought to recognize an HHV-7 late antigen and is thus used as an indicator of lytic infection (16).

**FIG 9:**
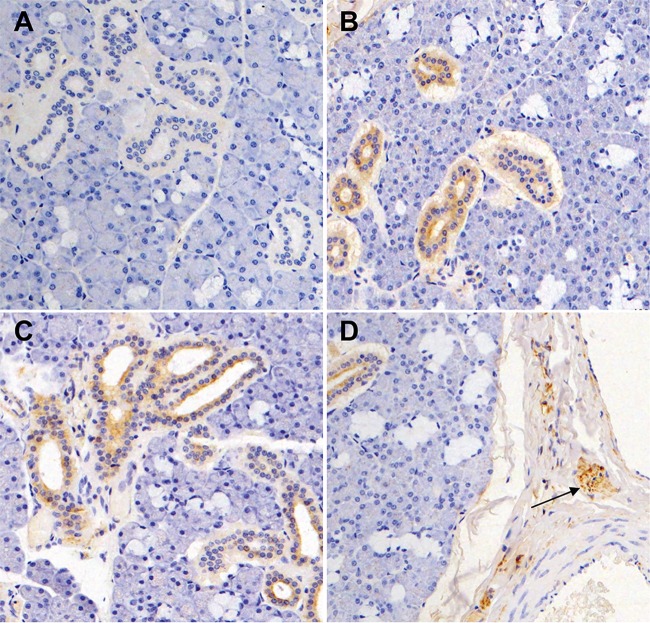
Immunohistochemical visualization of MneHV7 infection in macaque salivary glands. Sections from formalin-fixed salivary gland tissues were stained with the KR4 antibody (brown). (A) Salivary gland tissue with a low number of different MneHV7 transcripts detected by RNA-seq is KR4 negative. (B and C) Salivary gland tissues with intermediate (B) or high (C) levels of MneHV7 transcripts reveal specific cytoplasmic and occasionally perinuclear immunoreactivity in duct cells. (D) Positive staining is seen within peripheral nerve ganglia and nerve twigs (arrow).

We first tested whether the KR4 antibody showed cross-reactivity against MneHV7 proteins by IHC staining. In order to do so, we used sections from formalin-fixed paraffin-embedded salivary gland specimens available from our previously published study (17). To correlate IHC data with MneHV7 transcription levels, we selected specimens with viral gene expression profiles representative of those in the different salivary gland clusters observed in Fig. 8. Sections with transcripts of a large number of viral genes (macaque M03240, 63 genes), a medium number of viral genes (macaque M02393, 23 genes), or only a few viral genes (macaque M-2298, 8 genes) were obtained from macaque salivary gland tissues. As seen in Fig. 9A, we did not detect staining, using the KR4 antibody, of salivary gland tissue sections from macaque M02298, in which transcripts of only eight different viral genes (U28, U39/gB, U40, U42, U45, U48/gH, U56, and U81) were identified by RNA-seq. This demonstrated that the KR4 antibody does not cross-react with an endogenous macaque protein present in salivary glands. In contrast, we observed strong immunostaining in salivary gland sections from the two macaques in which a higher number of different viral transcripts were detected by RNA-seq (25 and 63 viral transcripts for macaques M02393 and M03240, respectively). Diffuse cytoplasmic immunoreactivity and, occasionally, perinuclear immunoreactivity were observed in salivary duct cells (Fig. 9B and C). This pattern of KR4 immunoreactivity is similar to what has been observed for HHV-7-infected human salivary glands, indicating that the KR4 antibody cross-reacts with a specific MneHV7 epitope in macaque tissue. Detection of a late viral protein in macaque salivary glands expressing a large number of viral transcripts but not in tissues with much more limited viral gene expression strongly suggests that MneHV7 undergoes active lytic replication in KR4-positive tissue specimens.

Like other tissues, salivary glands are innervated, and cell structures typical of nerve ganglia belonging to the peripheral nerve system are easily recognizable in tissue sections. Nerve ganglia are mostly composed of a cluster of neurons and glial cells. A very exciting observation was that, in addition to salivary duct cells, strong KR4-positive staining also occurred in multiple nerve ganglia present in salivary gland tissue sections from several MneHV7-infected macaques (Fig. 9D). This observation strongly suggests that, similar to HHV-7, MneHV7 is a neurotropic virus and has the ability to infect cells of the peripheral nervous system (PNS) in naturally infected macaques.

## DISCUSSION

In this study, we determined the complete unique genome sequence of the pigtailed macaque HHV-7 homolog MneHV7 together with a substantial portion of the direct repeat region found at both genomic termini using high-throughput sequencing of macaque saliva DNA. Detailed genomic analysis revealed a high degree of conservation between the simian virus and its human counterpart, HHV-7, at both the level of the genome structure and the sequence level. Thus, our observations demonstrate that MneHV7 is a new macaque roseolovirus homolog of HHV-7. Phylogenetic analysis confirmed that MneHV7 clustered with HHV-7 and other HHV-7 homologs discovered in great apes, creating a separate lineage among roseoloviruses. In addition, we characterized the MneHV7 transcriptome in macaque salivary glands using RNA-seq and confirmed the MneHV7 tropism for salivary duct cells, thus confirming the major role of this tissue in the MneHV7 life cycle.

Sequencing of the MneHV7 unique genome from macaque saliva at a mean read depth of 25 nucleotides revealed that the genomic structure, including the location and orientation of all open reading frames, was identical between the macaque virus and its human homolog, HHV-7. In addition, we found strong conservation between the MneHV7 and HHV-7 (RK strain) genome sequences, which showed a 64% genome-wide nucleic acid sequence identity. Importantly, the MneHV7 genome contains the ORF U55B, a gene that is present only in HHV-7 strains and that is lacking from HHV-6 genomes. In contrast, HHV-6-specific ORFs U22 and U96 had no gene homologs present in the MneHV7 genomic sequence. Other similarities included the numbers, locations, and degrees of conservation of splice sites in genes that have been predicted to be spliced in HHV-7 RK, suggesting that these genes may be similarly spliced in MneHV7. Other roseolovirus genome sequences currently available were obtained from isolates after several passages in cultured cells, which are likely to result in sequence variation, particularly in the DR regions. In contrast, the MneHV7 genome sequence that we reported here was obtained directly from a DNA template isolated directly from saliva collected from a naturally infected macaque. It will allow future assessment of viral genetic heterogeneity as additional sequences from other animals are made available.

The status of MneHV7 as a simian homolog of HHV-7 was further confirmed by phylogenetic analysis, which showed a close relationship between the known human HHV-7 strains and the novel MneHV7. Additionally, MneHV7 clustered most closely with recently discovered HHV-7 homologs in great apes, including gorillas and a number of chimpanzee species (18). All HHV-7 homologs clustered clearly separately from both human HHV-6A and HHV-6B species and HHV-6 primate homologs, indicating the presence of two distinct roseolovirus lineages that we provisionally named roseolo 1 and roseolo 2. Thus, viruses from each lineage have likely evolved simultaneously within their respective hosts.

The availability of the entire sequence of the unique region of the MneHV7 genome as well as large sections of the end-terminal DR regions allowed us to examine in detail sequence elements that are crucial for roseolovirus biology, such as DNA replication and integration. First, we examined the genomic ends by comparison of the sequences with those of the known human roseolovirus genomes. HHV-7 DNA replication is likely to involve a rolling-circle mechanism following circularization of the linear genome. In this circular form, two conserved sequence elements, Pac-1 and Pac-2, each of which is located at the ends of the genome, are brought into adjacent positions, allowing viral DNA cleavage and packaging to occur (40). Since we found that the MneHV7 end-terminal DR regions contain conserved Pac-1 and Pac-2 sequences at locations identical to their locations in HHV-7, a probable hypothesis is that MneHV7 relies on a mechanism similar to that relied on by HHV-7 for viral DNA replication and packaging. Additional elements present in the end-terminal DR regions of HHV-7 and located adjacent to Pac-1 and Pac-2 include two large telomeric repeat regions (T1, T2). The T1 and T2 regions are composed of reiterations of the TAACCC telomeric motif (41, 42). Homologous recombination between viral and chromosomal telomeric repeat sequences is thought to be the mechanism that allows viral integration into subtelomeric regions of host cell chromosomes. While viral genome integration into host cell chromosomes has been demonstrated for HHV-6 (43–45), it has not been documented for HHV-7. Nonetheless, the presence of highly conserved regions corresponding to the T1 and T2 telomeric repeats in the MneHV7 genome strongly suggests an important biological role for these genetic elements in the roseolovirus life cycle.

Based on the sequence similarity with the genomes of the available HHV-7 strains, we identified the putative oriLyt region for MneHV7 to be in the intergenic region between the U41 and U42 genes. Similar to the oriLyt in human roseoloviruses, the MneHV7 oriLyt consists of two highly conserved sequences with strong similarity to origin-binding protein 1 (OBP-1; box I) and OBP-2 (box II) binding sites separated by an AT-rich spacer element. A third putative OBP-binding site conserved between HHV-7 and HSV-1/2 but absent in HHV-6A and HHV-6B has also been reported (13, 46). Although this site is also conserved in the macaque herpes simplex virus homolog (macacine herpesvirus 1), we detected no sequence with significant similarity to that site in the MneHV7 oriLyt sequence. The absence of sequence conservation in the third OBP-binding site in the MneHV7 genome supports previous observations that this genetic element is not essential to the interactions between replication factors at the oriLyt.

OBP is encoded by the HHV-7 U73 gene, and the protein domains that interact directly with box I and box II in the oriLyt region have been characterized (13). The predicted MneHV7 OBP shares 69% amino acid sequence identity with the HHV-7 OBP amino acid sequence, while the domains that bind oriLyt share even higher levels of amino acid sequence identity (81% for the roseolovirus conserved domain within subdomain A and 84% for the herpesvirus OBP domain within subdomain B). When conservative amino acid changes are taken into account, the similarities between the same domains in the human and macaque viruses are 91% and 97%, respectively. Altogether, these observations strongly suggest that the MneHV7 OBP binds the MneHV7 oriLyt through conserved binding domains and that the MneHV7 DNA replication mechanism shares common features with its human roseolovirus counterparts. However, additional molecular studies are needed to characterize the precise sequence requirements for the interaction of MneHV7 OBP with the oriLyt.

Another feature that the MneHV7 genome shares with HHV-7 is the existence of major repetitive elements, R1 and R2. Little is known about the potential biological function of these repeat regions. Studies of HHV-7 and HHV-6 genome variability have found that the length of both the R1 and R2 regions varies between clinical viral isolates (47–49). The differences in lengths are due to a difference in the number of reiterated motifs in each repeat region. Since the MneHV7 genome sequence was established using DNA from saliva samples from a single infected animal and not a single cloned isolate, we were unable to determine variability in the repeat sequences. The locations of both repetitive elements in the viral genome were identical between MneHV7 and HHV-7. While the sequence and the length of the reiterated 105-bp motif in R2 were highly conserved between MneHV7 and HHV-7 (78 to 82% sequence identity with HHV-7 strains RK and JI), the sequence of the MneHV7 R1 motif was much shorter than that of the HHV-7 R1 motif (9 bp versus 67 bp) and showed no similarity with either the HHV-7 R1 sequence or the R1 sequences of other herpesvirus. The selective pressure to maintain repeated sequence motifs at these locations in the viral genome suggests that these genetic elements play an important biological role. Since no protein-coding ORF has been identified within the R1 or R2 region, it is tempting to speculate that their sequences could be transcribed as noncoding RNAs.

The availability of an annotated MneH7 genome sequence allowed us to investigate the viral gene transcription profiles in salivary gland tissues from nine infected macaques by RNA-seq. Although the first complete HHV-7 genome sequence was determined many years ago, viral gene transcription and temporal expression are still poorly characterized. Only recently, a study reported the detection of HHV-6B transcripts in a cohort with non-Hodgkin's diffuse large B cell lymphoma by using RNA-seq (25). The HHV-6B transcriptome showed a broad range of gene expression across the genome, indicative of lytic replication. Another recent report studied the temporal regulation of a few select HHV-7 genes and characterized their splicing patterns following *in vitro* infection of SupT1 lymphoid cells (16). PBMCs from healthy blood donors with a detectable amount of HHV-7 DNA were also analyzed for the presence of the same transcripts, but none of them could be detected. This supported the notion that HHV-7 latency in PBMCs is associated with very limited transcriptional activity. To our knowledge, HHV-7 gene expression profiling in infected tissue, including salivary glands, has not been documented.

We used high-throughput RNA sequencing to characterize the MneHV7 transcriptome in parotid gland specimens collected from a group of nine naturally infected macaques. We observed a statistically significant correlation between the overall number of normalized viral reads and the MneHV7 viral loads in saliva as well as salivary gland tissues. This strongly suggests that salivary glands are a natural anatomical site for MneHV7 replication and an important source of virus present in saliva. We also observed that the salivary gland tissue samples from the nine macaques had distinct viral gene expression patterns. Thus, the level of active replication of MneHV7 is different in each naturally infected specimen, which was also indicated by the variation in viral loads. To assess and visualize the dissimilarities between the different viral gene expression profiles, we performed a hierarchical cluster analysis. RNA-seq data showed separate clustering of salivary gland samples with high viral read counts versus salivary gland samples with lower viral read counts. As expected, the two animals with the highest MneHV7 loads in salivary gland tissue, as determined by qPCR, also had the highest read counts, as determined by RNA-seq, and broad gene expression profiles with expression of over half of all viral genes (55% and 57%, respectively). Such an expression profile is commonly associated with active lytic replication (25, 50, 51). Among the genes expressed in tissues with suspected active viral replication were several HHV-7 gene homologs that have been shown in HHV-7 to encode proteins that can potentially interfere with the host antiviral immune response. Among them, U12 and U51 encode functional beta-chemokine receptors (52, 53) and U21 is an immune evasin diverting major histocompatibility complex class I molecules to the lysosomal compartment (54–56). Additional experiments aimed at characterizing the expression of these immune-modulatory proteins in different naturally infected tissues are ongoing.

MneHV7 gene expression profiles in animals with lower viral loads showed a more limited expression of viral gene transcripts. Establishment of latency has been observed for HHV-6 following PBMC (57), monocyte/macrophage (58), and neuroglial cell (59) infection and is associated with a restricted pattern of viral gene expression. Although the mechanism of latency is still poorly understood, expression of the U94 gene has been associated with latency maintenance (60). Since there is no U94 gene homolog in either the HHV-7 or the MneHV7 genome, a viral gene expression pattern associated with latency has not been characterized yet. Further transcriptome studies are required to determine whether a restricted pattern of viral gene transcription in MneHV7-infected macaque tissue is indicative of viral latency.

Previous studies have characterized, using immunohistochemistry, the cell types in salivary gland tissues naturally infected with HHV-7 (9, 38). In these studies, a late HHV-7 protein recognized by the KR4 antibody was predominantly detected in the cytoplasm of ductal cells in human salivary gland tissue sections. Using the same antibody, we observed a similar immunostaining of duct cells from macaque salivary glands. This observation confirmed that the MneHV7 natural tropism closely mimics the tropism of its human homolog, HHV-7. In addition, specific immunostaining was observed only in sections from tissue specimens with high viral loads. Altogether, these observations support the hypothesis that the different MneHV7 expression profiles that we observed in salivary glands are representative of different MneHV7 reactivation levels that ultimately lead to active viral replication in salivary duct cells.

Roseoloviruses are considered to be mainly lymphotropic and neurotropic. The central nervous system (CNS) seems to be one of the reservoirs for persistent HHV-7 infection, since specific genomic sequences can be detected by PCR (61, 62). Nonetheless, HHV-7 reactivation measured by specific qPCR of blood samples is also associated with demyelinating diseases of the peripheral nervous system (PNS) (6). Most interestingly, we observed MneHV7-specific staining of nerve ganglia innervating the salivary glands. This strongly suggests that, similar to what has been reported for HHV-7, MneHV7 is neurotropic and that the PNS may be a natural reservoir for infection. Since the KR4 antibody recognizes a late viral protein, cells within the ganglia are likely to be lytically infected. Cytokine dysregulation is generally associated with roseolovirus reactivation in patients with acute illness (63–65). Cells in the peripheral nervous system express surface receptors allowing them to respond to cytokines (66, 67). Hence, local inflammation could be an important factor leading to MneHV7 active replication in the PNS. Additional studies aimed at determining the spread of MneHV7 infection in the PNS across different organs appear to be warranted.

Peripheral nerve ganglia are mainly composed of a cluster of nerve cells and glial cells called Schwann cells. Some of the cells stained in the nerve ganglia show a structure reminiscent of that of Schwann cells. Since HHV-7 reactivation is associated with demyelinating processes in humans (6), it is tempting to speculate that Schwann cells are susceptible to MneHV7 infection. MneHV7 lytic infection of Schwann cells could be associated with a decrease in myelin expression, resulting in demyelinating neuropathies. Additional studies are required to confirm the susceptibility of Schwann cells to MneHV7 infection and determine the resulting impact on myelin expression.

In summary, we present here a new animal model of HHV-7 infection based on the natural infection of pigtailed macaques with MneHV7. Our model is currently the first and only animal model available for studying HHV-7 infection and pathology. Determination of the MneHV7 genome sequence constitutes an advance in this model, allowing detailed comparative genomic analysis and in-depth transcriptome analysis using high-throughput sequencing techniques. The availability of naturally infected tissues offered us the opportunity to study the gene expression profiles of a simian homolog of HHV-7 in tissue specimens that would be difficult to obtain in human studies. Therapeutic strategies aimed at preventing roseolovirus reactivation are limited by an incomplete understanding of the mechanisms by which roseoloviruses establish infections and persist in tissue reservoirs with occasional reactivation. The macaque model of HHV-7 infection offers a unique opportunity to obtain critical insights into the cellular and viral factors promoting roseolovirus reactivation in natural reservoirs.
